# Localization of causal locus in the genome of the brown macroalga Ectocarpus: NGS-based mapping and positional cloning approaches

**DOI:** 10.3389/fpls.2015.00068

**Published:** 2015-02-19

**Authors:** Bernard Billoud, Émilie Jouanno, Zofia Nehr, Baptiste Carton, Élodie Rolland, Sabine Chenivesse, Bénédicte Charrier

**Affiliations:** Sorbonne Université, UPMC University Paris 06, CNRS, UMR 8227, Integrative Biology of Marine Models, Station Biologique de RoscoffCS 90074, F-29688, Roscoff cedex, France

**Keywords:** macroalga, mutant, genetic locus, positional cloning, NGS-based mapping, SNP mapping, SSR

## Abstract

Mutagenesis is the only process by which unpredicted biological gene function can be identified. Despite that several macroalgal developmental mutants have been generated, their causal mutation was never identified, because experimental conditions were not gathered at that time. Today, progresses in macroalgal genomics and judicious choices of suitable genetic models make mutated gene identification possible. This article presents a comparative study of two methods aiming at identifying a genetic locus in the brown alga *Ectocarpus siliculosus*: positional cloning and Next-Generation Sequencing (NGS)-based mapping. Once necessary preliminary experimental tools were gathered, we tested both analyses on an Ectocarpus morphogenetic mutant. We show how a narrower localization results from the combination of the two methods. Advantages and drawbacks of these two approaches as well as potential transfer to other macroalgae are discussed.

## To which extent can current forward genetic approaches be profitable to macroalgal developmental studies?

The development of macroalgae has been scarcely investigated, especially with functional approaches. Numerous growth patterns at the embryo stage or during branching were reported, either at the microscopic scale by thorough observation of cell and tissue organization (Fritsch, 1945) or at a larger scale in various environmental conditions (Littler et al., 1983; Hanisak et al., 1988; Steneck and Dethier, 1994; Balata et al., 2011). This knowledge is crucial preliminary to the study of the development of any organism. Some modeling studies came to nicely reinforce our understanding of how macroalgae develop, especially by proposing rules based on cellular events, which, when re-iterated several times, could account for the overall algal morphology (Lück et al., 1999; Billoud et al., 2008). However, these approaches are devalued by their incapacity to decipher the molecular events underlying these morphological processes. Mutant analysis, which is one of the most powerful approaches used in functional studies on all kinds of organisms, allows to uncover new biological mechanisms *de novo*, *i.e*., without leaning on previous knowledge. Several morphological mutants of macroalgae such as the green *Ulva*, the red *Gracilaria* and the brown *Ectocarpus* have been generated by UV or chemical mutagenesis and summarily studied at the genetic level (reviewed in Charrier et al., submitted).

Beside the formulation of genetic pathways based on epistatic relationship, the molecular identification and characterisation of genes involved in the modified biological process remains the main goal of mutant analyses.

**Figure 1:**
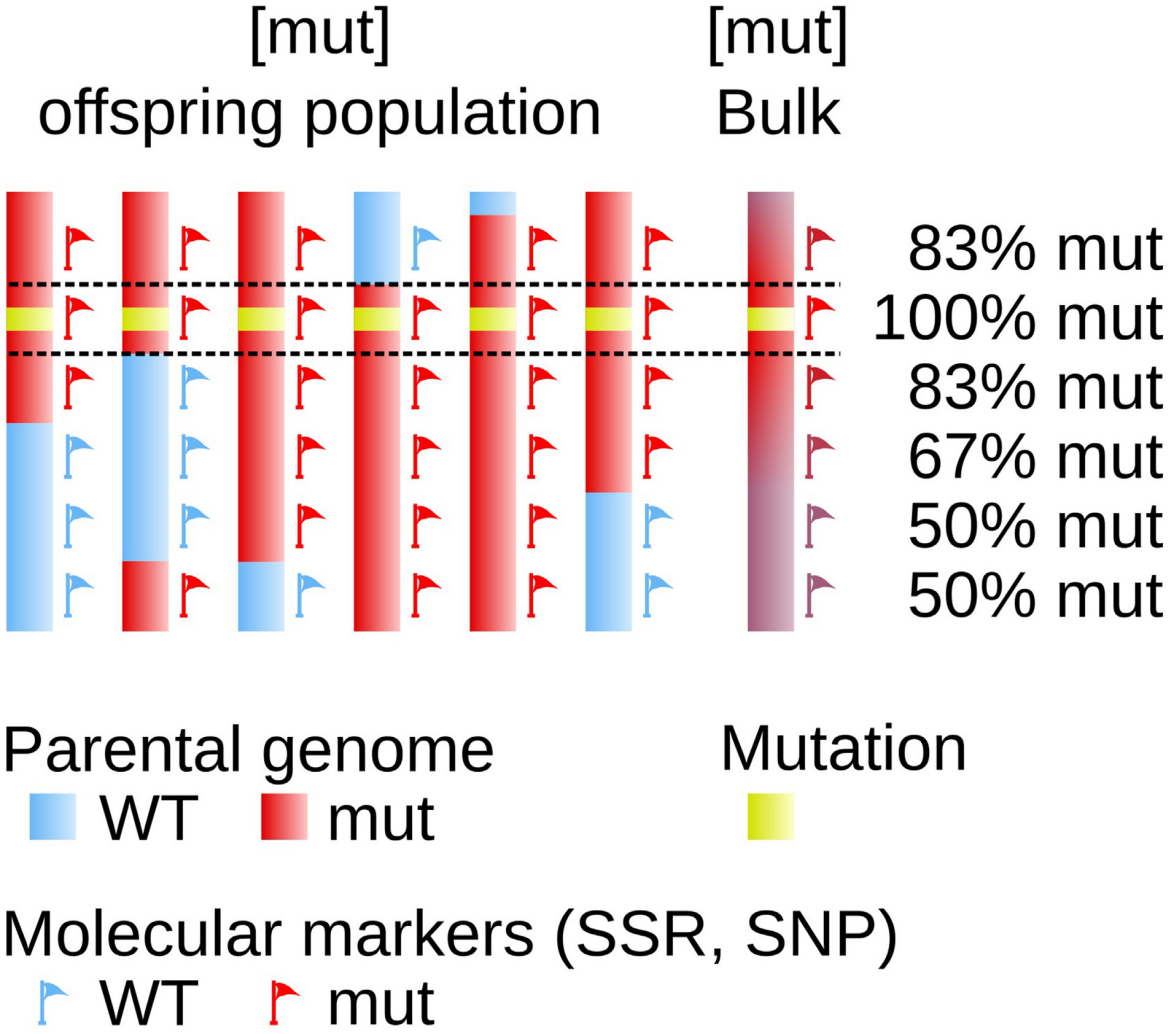
**Genetic linkage determines the physical position on the genome**. The genome of 6 offspring individuals is displayed as a simplified representation of a larger offspring population. One parent (red) carries a mutated locus (yellow box). It shares with the sexual partner (blue) some genomic polymorphic markers spread over the genome, used as molecular markers allowing to indicate position on the genome (red and blue flags). The genetic linkage observed after meiosis between the mutation and one given molecular marker is dependent on the genetic distance, and to some extent on the physical distance, between these two loci. The farthest the two loci, the highest the probability for them to be segregated apart in the offspring, as a result of crossing-overs taking place during meiosis.

The availability of transposon- or transgene-tagged mutants allows to fairly easily fish out the mutated gene. But when this type of approach is not possible, finding the mutated gene becomes a more laborious work equivalent to be looking for a needle in a haystack. Several methods, based on similar principles, have been developed. They consist in measuring the genetic linkage between the mutated locus and a set of known polymorphic markers. The principle of genetic linkage, which requires the analysis of an offspring population generated from the mutant organism, has been established more than one century ago (Sturtevant, 1913) and was successfully used in developmental studies in animals and plants (Vollbrecht and Sigmon, 2005). It relies on the frequency of the recombination events (cross-overs) during the meiotic stage: the closest to the mutation one marker, the lowest the number of recombination between these two loci in the progeny (Figure 1).

In the past, these markers were visual, but in the last 20 years, different kinds of molecular markers were developed, all based on genomic sequence differences between the genetic background of the mutant organism and the sexually compatible partner used to generate the offspring. They ranged from single nucleotide polymorphism (SNP) in sequences corresponding to restriction sites (usually hexamers) to the length of microsatellite (or Single Sequence Repeat SSR, also called SSLP for “simple sequence length polymorphism”) sequences and more recently to SNP in any location. The advent of the PCR technique and the analysis of the pooled segregating populations instead of individuals [“Bulk segregant analysis”, see Michelmore et al. (1991) and Quarrie (1996)] eased the characterisation of this molecular polymorphism: methods called “Amplification of Fragment Length Polymorphism” (AFLP) for the restriction sites, “SSR amplification” for the microsatellites, and “Snapshot” for the SNP were developed in a close past (Mueller and Wolfenbarger, 1999; Lukowitz et al., 2000; Jander et al., 2002). These methods are suitable for the identification of both single-locus and multi-locus (*e.g.*, QTL) genetic determinants (Gebhardt et al., 2005; Matsubara et al., 2014).

More recently, several NGS-based mapping approaches were developed in land plants and metazoa: SHOREmap (Schneeberger et al., 2009) and New Gene Mapping or NGM in Arabidopsis (Austin et al., 2011) and in metazoans (Zuryn et al., 2010; Bowen et al., 2012). They involve batch sequencing of offspring populations, and are based on the conservation of the mutated locus in all mutant individuals of the offspring, while its genomic environment is fluctuating as a result of cross-overs during meiosis. More recently, Tabata et al. (2013) localized Ethyl-Methane-Sulfonate (EMS)-induced mutations in rice using a combination of classical genetics and low-coverage NGS. Laitinen et al. (2010) and Uchida et al. (2011) managed to identify a mutation by NGS-based mapping in a non-referenced genome of *Arabidopsis thaliana*, displaying 0.5% of genomic sequence difference with the referenced accession Columbia. These approaches were also successful in larger genomes like rice (reviewed in Lee and Koh, 2013).

**Figure 2:**
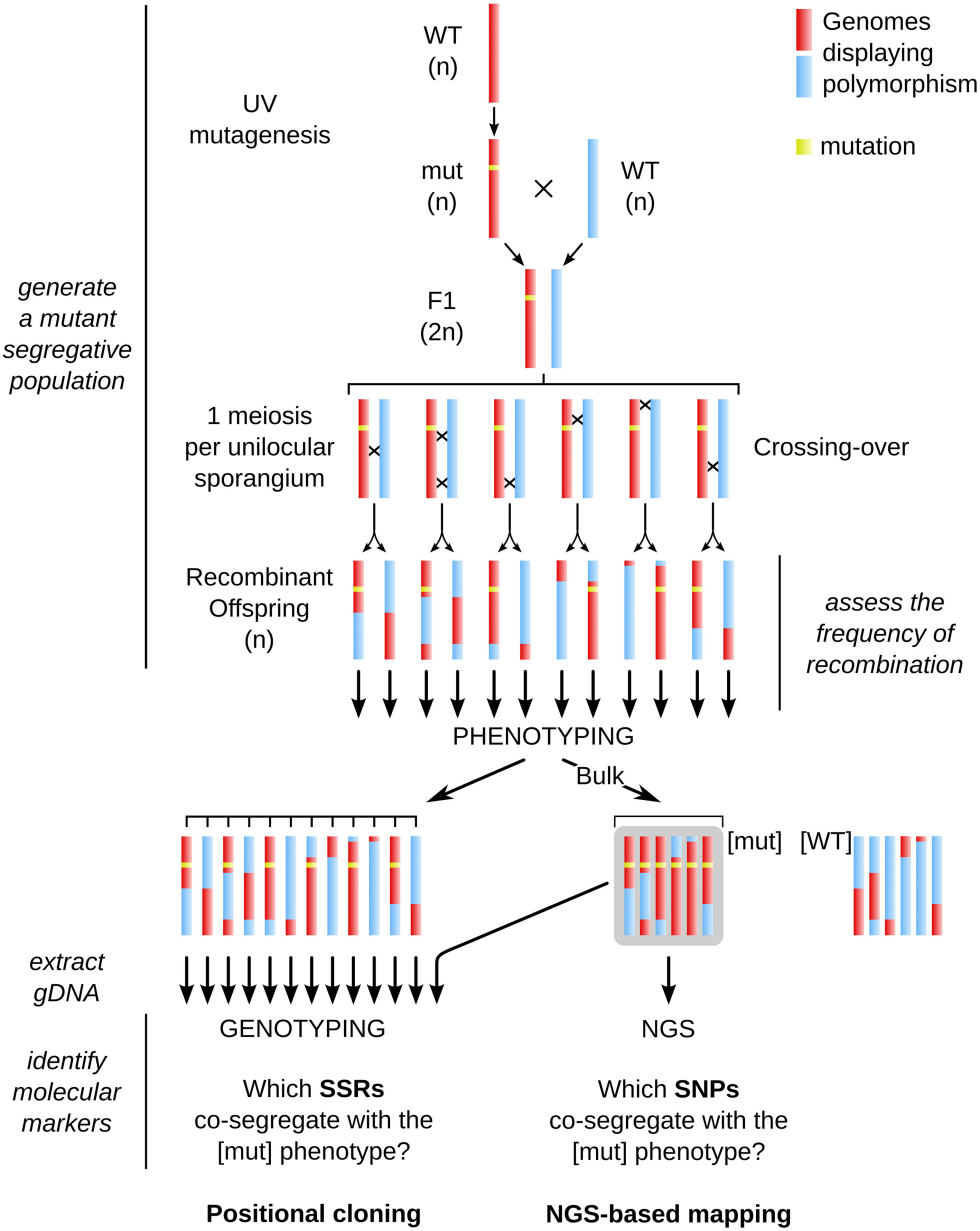
**General chart of the different steps necessary for achieving positional cloning and Next-Generation Sequencing (NGS)-based mapping in Ectocarpus**. The whole procedure is based on the generation of a mutant segregative population from two parents, the occurrence of recombination events at the meiotic stage, phenotyping of each offspring individual, extraction of genomic DNA from the [mut] offspring, on which either NGS (for NGS-based mapping, right side) or PCR amplification of molecular markers (genotyping with SSR markers in this study, left side) were carried out. Rough identification of the *mut* locus by genotyping was first performed on a bulk population, and then fine mapping was carried out on individual gDNA extractions. Note that phenotyping and subsequent genotyping are performed in haploid individuals (parthenosporophytes, see Figure 3 for details on the life cycle of Ectocarpus). See legend of Figure 1 for color code.

As macroalgal morphological mutants mentioned above were not tagged, the identification of the mutated locus requires approaches such as positional cloning or NGS-based mapping. Here we report the experimental procedure and the results obtained when using these two approaches to identify a mutated locus in the brown alga *Ectocarpus siliculosus*. The different steps are summarized in Figure 2.

## Specific knowledge and tool requirement prior to localisation of a mutant locus

### Generating a segregative population

**Figure 3:**
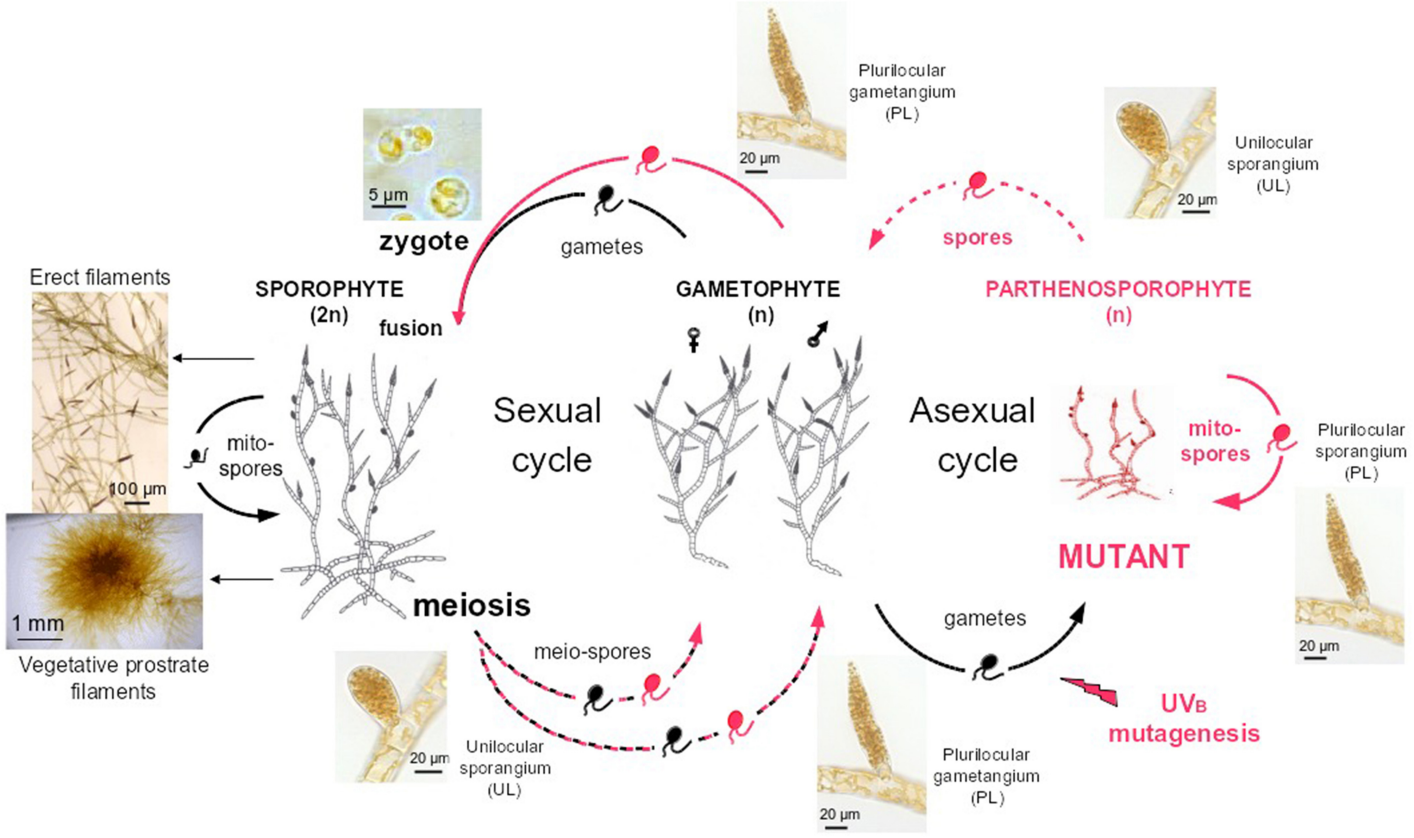
**Life cycle of *Ectocarpus siliculosus***. Both Ectocarpus sexual and asexual life cycles are displayed. Gametes (right side) released from WT male gametophytes (strain Ec32) are mutagenised by UVB irradiation (Le Bail and Charrier, 2013). Upon a phenotypic screen, mutants (red) are propagated 5 times asexually, by successive release of mitospores from plurilocular sporangia. Spores regenerate a mutant gametophyte, which was crossed with a WT sexual female partner (Ec568). All mutations obtained so far in Ectocarpus were shown to be recessive in the diploid sporophyte (left side) (Peters et al., 2008; Coelho et al., 2011; Le Bail et al., 2011). Meiosis generates male and female mutant or WT gametophytes. Dashed lines stand for meiospore release from unilocular sporangia, plain lines for spore or gamete release from plurilocular sporangia and gametangia respectively. Prostrate filaments and erect filaments characteristic of the general morphology of *Ectocarpus siliculosus* sporophyte are also displayed (left part).

Wild type *Ectocarpus siliculosus* strains (Peters et al., 2010) Ec32 (accession CCAP 1310/4, male) and Ec568 (accession CCAP 1310/334, female) were cultivated according to published procedures (Le Bail and Charrier, 2013). Mutant named therein “mut” was obtained from UV-B irradiation of *E. siliculosus* Ec32 gametes according to Le Bail and Charrier (2013). Compared to the WT organism, it displayed an impaired branching pattern and an early differentiation of upright filaments. The phenotype was shown to be stable after five generations of parthenogenetic reproduction (Figure 3).

The mutant was crossed with the sexual partner Ec568 as described in Le Bail et al. (2011) To some extent, these two strains display molecular polymorphism as shown by Heesch et al. (2010). Once mature, the F1 sporophyte differentiates both plurilocular and unilocular sporangia (Figure 3). Each unilocular sporangium is subject to a single meiotic event (Knight, 1930). As the result of post-meiotic successive mitoses, each sporangium releases ~ 100 meiospores which germinate into gametophytes (Müller, 1975). A segregative population was generated from the F1 individual by isolating one single haploid gametophyte from one single unilocular sporangium. Phenotyping and subsequent work is carried out on the haploid parthenosporophytes generated from the non-fertilized gametes of each gametophyte and itself able to asexually reproduce after release and germination of its mitospores [asexual cycle on the right of Figure 3, or Charrier et al. (2008)]. The generated population was stored at 4°C in dim light for up to 2 years.

### Genome assembly, genetic map and recombinant frequency

**Figure 4:**
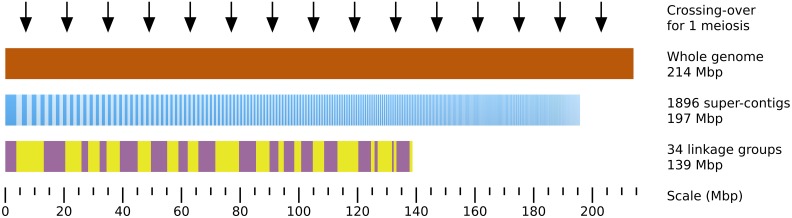
**Topological genomic information**. An overview of the genomic and genetic tools available to date on *Ectocarpus siliculosus* is presented. 92% of the total genome size estimated by flow cytometry to 214 Mbp (brown) (Peters et al., 2004) is covered by 1896 super-contigs (sctg, dark and light blue vertical bars, ordered by decreasing length, from 3746 to 2 kb). The 34 linkage groups (LGs) have been constructed by stringing sctgs together, based on their genetic linkage in an offspring population of 60 individuals (Heesch et al., 2010). The LGs are arranged from LG01 to LG34 (alternating purple and yellow boxes). The average distance separating 2 crossing-overs calculated in the present study in an offspring population composed of 91 individuals is 14 Mbp (black arrows).

Localizing a mutated locus on the genome requires some prior topological information. Previous studies showed that *Ectocarpus siliculosus* contains ~ 25 chromosomes, and its genome size is estimated to 214 Mbp (Peters et al., 2004), making average chromosome size of 8.56 Mbp (Figure 4). Sequencing of the genome allowed to assemble 197 Mb in 1896 super-contigs with sizes ranging from 2 to 3745.6 kb (average size = 104 kb, N50 = 497 kb) (Cock et al., 2010). A genetic map was built by performing segregation analyses of SSR markers present on the super-contigs (sctgs) and shown to be polymorphic between the two Ectocarpus strains used for the cross. It allowed to agglomerate the sctgs into 32 linkage groups (LGs) covering 65% of the genome only *i.e.*, 139 Mbp (Heesch et al., 2010), making average LG size of about 4.05 Mbp. Actually, a high level of heterogeneity in the size of the LGs is observed (from 578 to 9.36 Mbp, sd = 2.09 Mbp). In addition, LGs are not complete as sctgs assembled in one given LG do not overlap with each other. Moreover, the orientation of sctgs is unknown in most cases. Therefore, in spite of this unique breakthrough in the genome structure of a brown alga, the topological information remains partial (Figure 4).

Besides these topological information, we undertook experiments to assess the occurrence level of genetic recombination during meiotic events. Indeed, the lowest the frequency of crossing-overs occurrence, the largest the population of offspring individuals to analyse, and assessing the size of the required offspring population is a crucial parameter to determine before initiating these forward genetic approaches. Therefore, we undertook genotyping of available SSR markers (Heesch et al., 2010) on an offspring that we generated from the cross Ec568 × Ec32.

#### Amplification of Ectocarpus SSR markers

Genotyping was carried out by PCR amplification of SSR markers. Genomic DNA was extracted from lyophilised and ground Ectocarpus thalli according to the protocol of the NucleoSpin 96 Plant II kit (Macherey-Nagel). Amplification of SSR markers was then performed by PCR on 10 ng of purified gDNA using 0.5 u of GoTaq enzyme (Promega), 0.2 mM final concentration of dNTP and 2 mM MgCl_2_ in a 50 μL reaction volume for 5 min at 95°C followed by 12 cycles corresponding to 30 s at 95°C, 1 min at 65°C to 53°C (touchdown) and 30 s at 72°C, and 25 cycles corresponding to 30 s at 95°C, 1 min at 53°C and 30 s at 72°C. Oligonucleotides framing the microsatellite were either chosen among the 406 polymorphic SSR reported by Heesch et al. (2010) or designed *de novo* from the *E. siliculosus* genomic sequence (Cock et al., 2010) using the WebSat software (Martins et al., 2009). Forward oligonucleotide sequences were 5′-extended with the M13 oligonucleotide sequence (5′-TGTAAAACGACGGCCAGT-3′) to allow 5′ labeling of PCR product as described in Schuelke (2000). Final concentrations of 0.1 μM forward, 0.4 μM reverse and 0.4 μM FAM-labeled-M13 (Applied BioSystem) were used in the PCR reaction. The PCR reaction was completed by 10 min at 72°C and stored at 4°C. 2 μL of PCR products were then denatured by addition of 9.5 μL formamide and 0.5 μL of size ladder GeneScan 500-Liz (Applied Biosystem) and run on an ABI Prism 3130XL capillary sequencing machine (Applied Biosystem). Length polymorphism was analyzed with the GeneMapper version 4.0 (Applied Biosystem) software.

#### Cross-over frequency between the two Ectocarpus strains Ec568 and Ec32

In order to assess the frequency of cross-overs (COs) in this species, SSR genotyping analysis was performed on 91 individuals of the offspring from the cross Ec568 × Ec32. Each offspring individual was produced from a single meiotic event (*i.e.*, from a single unilocular sporangium; Figure 3), with 91 microsatellite markers covering 32 LG (from 1 to 5 SSR depending on the size of the LGs) (Supplemental Table 1). In this set of data, recombinations occurred in average 10.6 times per individual. As LGs cover only 65% of the total genome, one can speculate that the total number is 15 COs per individual in average over the whole genome. Therefore, considering the size of the genome (214 Mbp), COs occur every 14 Mbp in average, and 1 cM corresponds to 140 kb. This value, computed using 91 markers in a population of 91 individuals, is higher than the average value calculated by Heesch et al. (2010) on an offspring population of 60 individuals and using 406 SSR (54 kb.cM^−1^). The difference observed might be due to the lower number of markers in our experiment, which precludes displaying additional cross-overs. In addition, some markers were not efficiently amplified in our experiment (Supplemental Table 1). Therefore, the average physical distance corresponding to 1 cM is probably below 100 kb. Indeed, our calculation on LG08 (see below) resulted in a value of 76 kb.cM^−1^. In all cases, this distance is lower than what was calculated in *Arabidopsis thaliana* (average = 250 kb.cM^−1^, Lukowitz et al., 2000), a plant species in which most of the technological improvements related to the identification of mutations were achieved. Under direct dependence to the CO density, the size of the offspring population to analyse should be calculated so that the genomic window generated by the recombination events is large enough to allow identification of several molecular markers, but narrow enough to contain a reduced number of genes. Our data allow to predict that in Ectocarpus, 200 offspring lines should be necessary to frame the mutation within a 50 kb region, and molecular markers will have to be numerous enough in this genomic region so that this window can be spotted.

**Figure 5:**
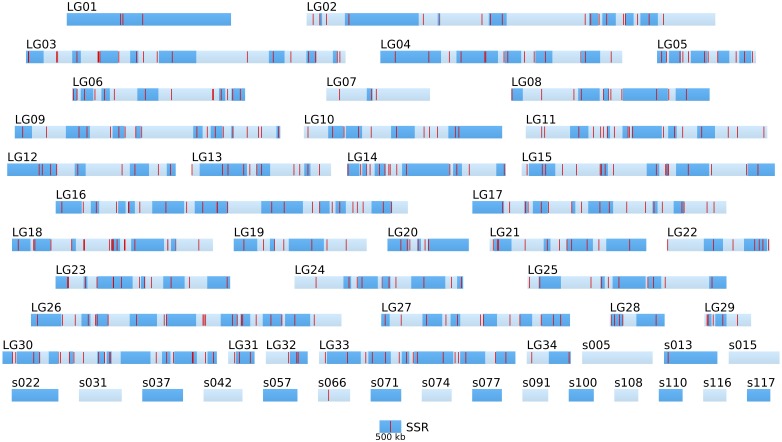
**Positional information: distribution of SSR markers in the genome of the two parental strains**. SSR markers (red bars) identified by Heesch et al. (2010) are positioned on the 34 linkage groups (LGnn) and super-contigs of length >500 kb (snnn). LGs are composed of several super-contigs (dark and light blue, in alternation). Super-contigs and LGs are drawn to scale. The sctg orientation has been taken into account when known.

Hence, the success of positional cloning and/or NGS-based mapping relies on a high density of molecular markers over the whole genome. The density of the currently available SSR in the genome of Ectocarpus is variable and remains altogether low (Figure 5). Therefore, the identification of SNP markers was undertaken.

### Identification of ectocarpus SNP markers

SNP markers are usually identified by sequence comparison of the genome sequence of two individuals from different genetic background. This was performed by NGS sequencing of genomic DNA from the two parental strains Ec32 and Ec568.

#### DNA preparation and Next Generation Sequencing (NGS)

Prior to sequencing, genomic DNA was prepared from bulk offspring Ectocarpus individuals. 1 g of dry and frozen tissue was homogenized with clean sand in liquid nitrogen. The powder was then agitated for 30 min in 10 mL extraction buffer (Tris-HCl pH 7.5 100 mM, NaCl 1.5 M, CTAB 2%, EDTA 50 mM, DTT 7.5 g.L^−1^ extemporaneously). Cellular debris and proteins were extracted twice with 1 volume of chloroforme:isoamyl 4:1 and 20 min centrifugation at 10,000 g at 20°C. RNAs present in the supernatant were precipitated with 2.4 M LiCl and 1% β-mercaptoethanol overnight at −20°C and eliminated in the pellet by 1 h centrifugation at 10,000 g at 4°C. DNA was then precipitated with 50% isopropanol and 0.3 M sodium acetate pH 5.2 30 min at 4°C, recovered in the pellet after 30 min 10,000 g centrifugation at 4°C and dissolved in 400 μL of sterile ultrapure water. The DNA was precipitated again with 0.3 M sodium acetate pH 5.2 and 2.5 volumes ethanol 100% for 30 min at 4°C. The sample was then centrifuged for 30 min at 10,000 g and the DNA pellet was washed with 75% ethanol. The dry pellet was dissolved in 2 mL of TE buffer (Tris-HCl pH 8.0 10 mM, EDTA 1 mM) with 5.4 M CsCl (density 1.66) and 37 μg.mL^−1^ Hoeschst (Sigma n°33258 bis-benzimide). DNA was centrifuged 24 h at 90,000 g at 25°C. DNA was viewed under UV light and extracted with a needle. Hoechst dye was eliminated from the DNA solution by five washing steps with 1 vol butanol saturated with TE buffer. Pure genomic DNA was alcohol-precipitated again, dissolved in 50 μL of sterile ultrapure water and its concentration was measured with NanoDrop 2000 (Thermo Scientific). 30 μg of CsCl-purified genomic DNA was sent to the MGX platform (http://www.mgx.cnrs.fr/) for sequencing using the short read technique on a HiSeq2000 (Illumina) sequencer.

#### In silico analyses

**Figure 6:**
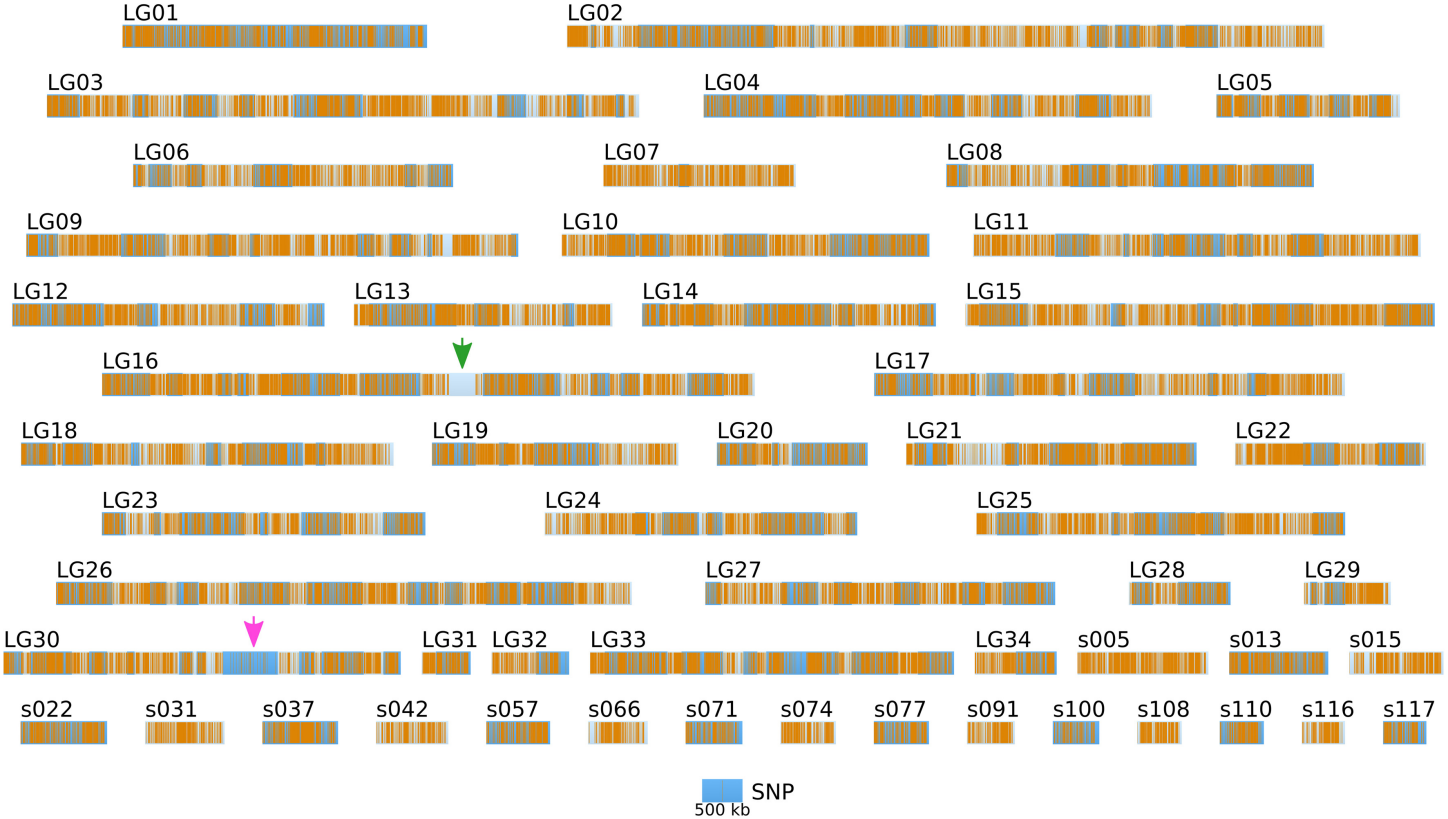
**Positional information: distribution of SNP markers in the genome of the two parental strains**. SNP markers (orange bars) identified from NGS sequencing data from Ec32 and Ec568 are displayed on the LGs and sctgs. See Figure 5 for further details about the legend. Green arrow, EsV1; Pink arrow, sex locus.

Short reads were trimmed using sickle version 1.33 (Joshi, 2011), leaving a total number of ~ 85 millions reads per strain. SNPs were directly inferred from the Ec568 and Ec32 trimmed short reads using the DiscoSNP software (Peterlongo et al., 2010) with kmers of size *k* = 31 and a minimal coverage *c* = 4. Then, each SNP was mapped onto the *E. siliculosus* genome by searching the optimal match of its surrounding sequence (the 61-mer centered on the SNP), using BLASTN 2.2.28+ from the ncbi-blast+ package (Altschul et al., 1990; Camacho et al., 2009), in ungapped mode, with a minimum identity of 96.7 (at most 2 unmatched nucleotides, including the SNP, among 61). Only unique matches were retained. By comparing the two parental sets of reads, 291274 SNPs could be identified, among which 83150 were purine-purine replacements, while 83048 were pyrimidine-pyrimidine replacements. As shown in Figure 6, the SNP density between the two parental strains Ec32 and Ec568 appeared globally variable at the scale presented, 147.10 ± 77.5 in a window of 200 kb. Two regions present a markedly lower SNP density than the average. (1) a region of ≈350 kb in the super-contig 52 (part of the LG16, green arrow) is devoid of SNPs and (2) a region of ≈900 kb comprising the super-contigs 68 and 285 (parts of the LG30, pink arrow) has a lower SNP density than the average. In both cases, the SNP depletion does not point out a lack of variability in these regions. On the contrary, mapping the short reads from strain Ec568 to the Ec32 genome resulted in only 69.9 reads matched/kb in sctg_52, and 17.0 reads matched/kb in sctg_68. As the average density of short reads in the rest of the genome (estimated on the 115 other super-contigs of length >500 kb) is *m* = 158.4 with a standard deviation sd = 23.8, the matching efficiency for sctg_52 and sctg_68 appeared to be 3.7 × sd and 5.9 × sd lower than the average, respectively. This shows that less (if any) SNPs are detected in these regions because their high variability prevents the identification of what discoSNP names a SNP: a region of 61 consecutive residues among which only the central base differs between the two strains. What lacks in these highly variable regions is not the differing base, but the 30 consecutive identical ones on each side. In addition, a closer examination of these two regions allowed to understand why they are so highly variable. The region of sctg_52 where no SNP could be identified corresponds to an insertion of the phycodnavirus ESV-1 (Delaroque and Boland, 2008). The high variability of virus copies in different strains thus explains the mapping and SNP results. Likewise, transcriptome studies showed an apparent low expression level in divergent strains for most of the genes located in this region (Dittami et al., 2011). The super-contigs 68 and 285 correspond to the sex-determining region (SDR) of *E. siliculosus*, as was first suggested by transcriptome analyses (Dittami et al., 2011), then confirmed by genetic mapping (Ahmed et al., 2014). Noteworthy, Figure 6 takes into account the improvement of the genetic map provided by Ahmed et al. (2014) over Heesch et al. (2010), by incorporating sctg_68 into LG30, as a result of their study on the SDR. However, the low density of SNPs was also readily detectable on sctg_68 alone, when this super-contig was analyzed apart from LG30 (not shown). As a conclusion, our data showed that a simple comparison between a male and a female strain genome sequences provides another, independent mean of identifying the SDR, which is both fast and easy to handle.

Altogether, the density of SNPs on the LG and long sctgs is far much higher (in average 1 SNP every 1.36 kb) than the density of the SSR markers (Figure 5).

## The mutant *mut* as a case study

Once these preliminary knowledge and tools in hand, we carried out both a positional cloning and an NGS-based mapping analysis as a pilot study. A mutant displaying an altered developmental phenotype compared to the WT, that we named *mut* for this study, was generated as described in the previous section. From the cross Ec568 × *mut*, a segregative population composed of 200 offspring individuals was generated. From this parthenosporophyte population, 48 [mut] individuals from independent meiotic events were isolated. They were either independently genotyped, or pooled for both SSR bulked genotyping (Positional cloning) or bulk sequencing (NGS-based mapping) (Figure 2). Genomic DNA was prepared as described in Section Specific knowledge and tool requirement prior to localisation of a mutant locus.

### Positional cloning

**Figure 7:**
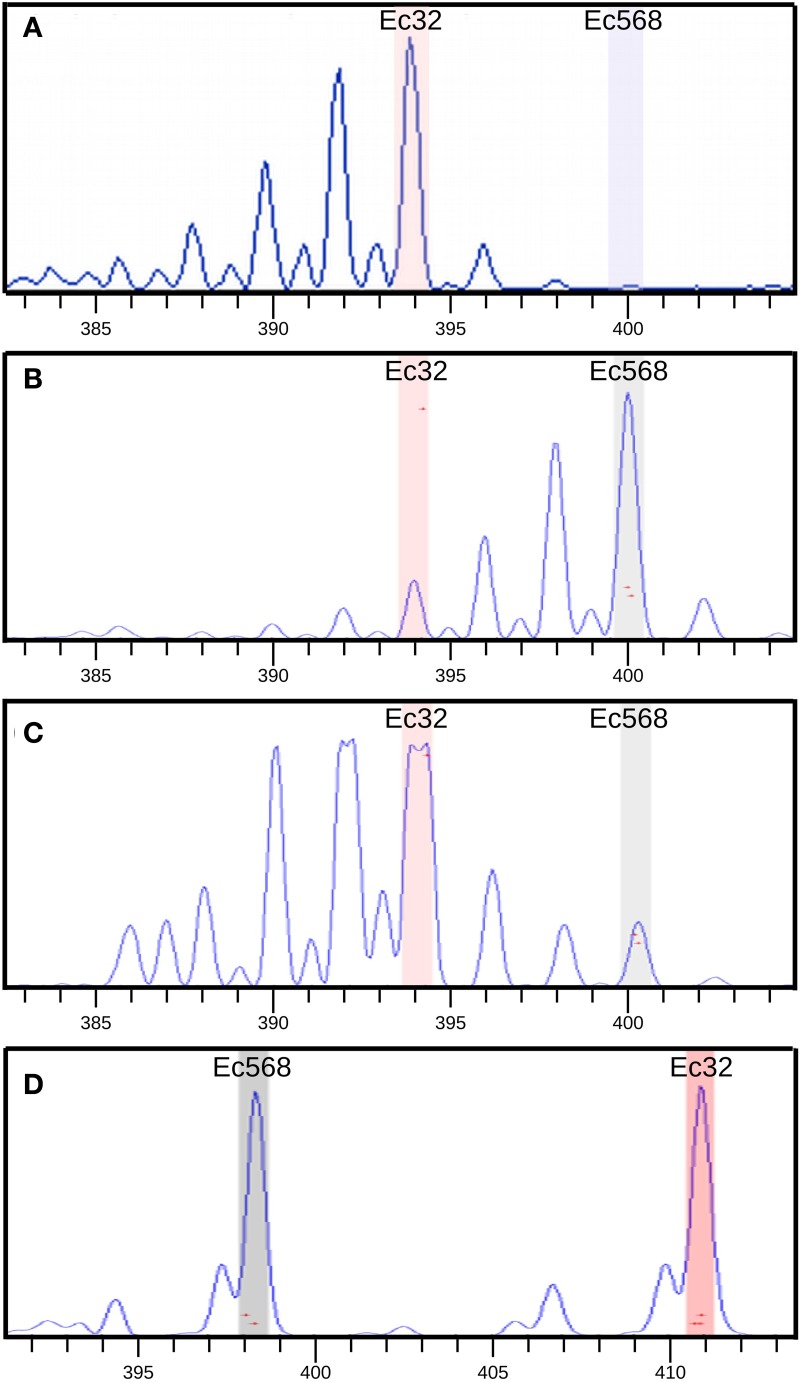
**SSR amplification in [mut] bulked gDNA**. Bulk amplification data were compared with amplification of two controls: Ec32 gDNA and Ec568 gDNA. Capillary electrophoresis profiles of the SSR amplification are shown. Size (nt) of PCR products is indicated in “x”, the amount of PCR product, relative to the height of the amplification curve is indicated in the “y”-axis (based on the measured fluorescent signal). **(A)** SSR M010_4 amplification on Ec32 gDNA showing an amplification curve corresponding to the Ec32 allele (“a” in Table 1), **(B)** M010_4 on Ec568 gDNA showing the curve corresponding to the Ec568 allele (“b” in table 1). Smaller peaks (on the left) correspond to stutters which are due to some partial amplification of the microsatellite sequence by the Taq polymerase. **(C)** M010_4 on bulked [mut] gDNA showing the prevalence of the Ec32 allele compared to the Ec568 allele. Note that the detection of the fluorescent signal corresponding to the Ec32 allele is saturated (forked peak). The genomic region carrying this allele is physically linked to the *mut* locus. **(D)** Amplification of SSR M_072 (LG11) on bulked [mut] gDNA, showing the presence of the two alleles in equal amount. The region carrying this allele is not linked to the *mut* locus.

In a first step, bulked DNA was genotyped with 52 SSR markers, equally distributed on the 34 LGs. A single SSR was chosen for small LGs, and up to 3 for the largest ones (*e.g.*, LG02). During meiosis, all LGs but that carrying the mutation should segregate randomly from the mutated locus and hence, in this bulked gDNA, these LG-specific SSR markers should display PCR signals corresponding to both the Ec32 (*mut* genetic background) and the Ec568 genomes in equal quantities (Figure 1). For SSR markers present on the LG carrying the mutated locus, a bias toward a stronger signal for the Ec32 specific SSR should be observed. Genotyping results showed that all SSR except M010_4 on LG08 displayed an equal distribution in the bulked DNA (Figure 7). In contrast, M010_4 amplification showed an higher amount of Ec32-specific SSR compared to Ec568-specific SSR, indicating that this marker is in the vicinity of the mutation *mut*. Therefore, LG08 likely carries the mutated locus.

**Figure 8:**
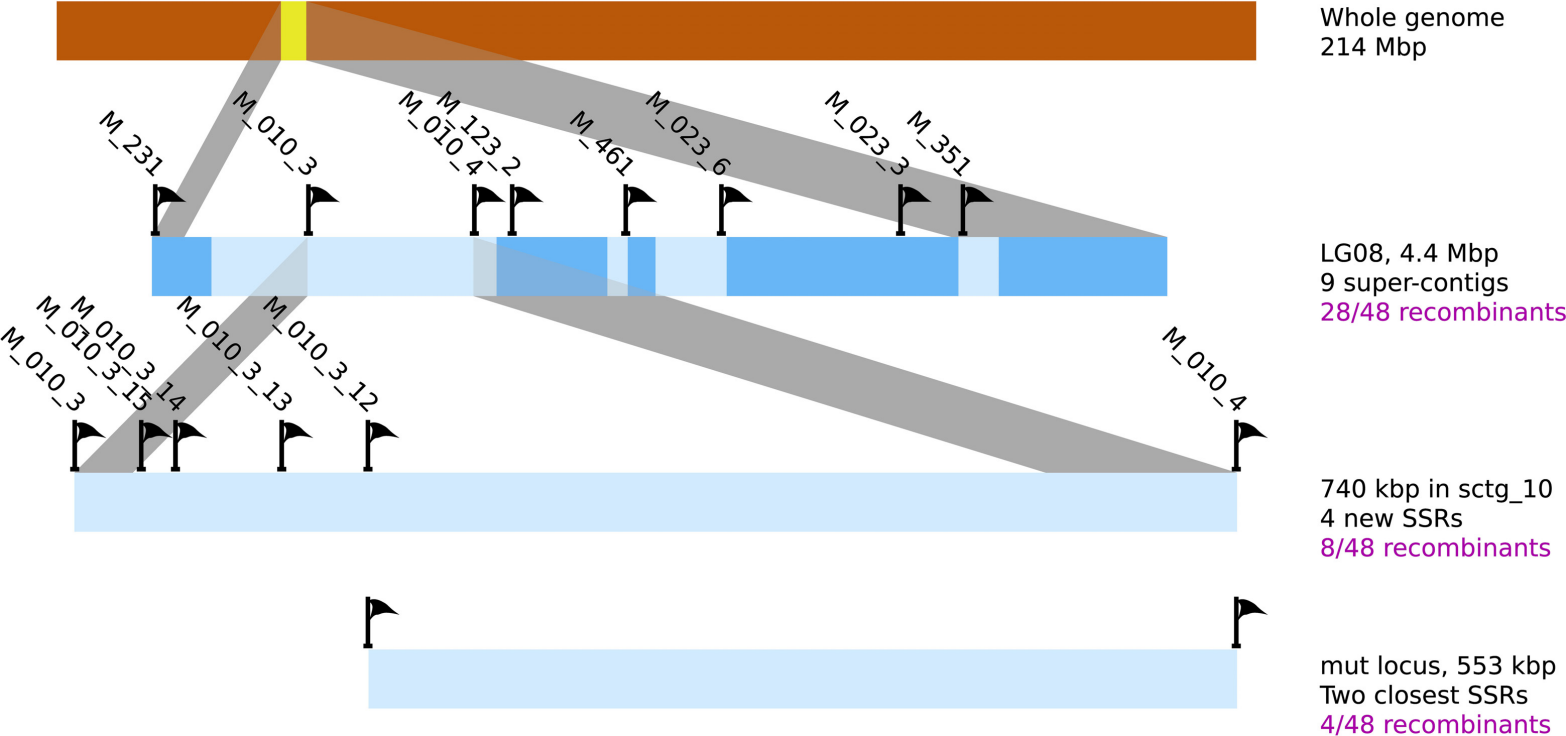
**Position of the mutated locus inferred from positional cloning**. The progressive selection of a genomic region containing the *mut* locus from the LG08 is illustrated. Sctgs composing this LG08 are displayed as well as the position of the molecular markers (SSR). Additional SSR markers were designed on sctg_10 (see Supplementary Table 2 for corresponding oligonucleotide sequences and amplification product sizes). Among 10 SSR sequences in the region between M010_3_12 and M010_4, none displayed polymorphisms between Ec32 and Ec568. The number of recombinant individuals from the offspring is indicated in the right margin.

In a second step, and in order to support the previous result, a fine mapping was separately conducted on each [mut] individuals displaying a recombination event in this region. 48 individuals were tested with 8 SSR, present in 6 sctg composing LG08 (2 SRR for the largest sctg, *i.e.*, sctg_10 and sctg_23). From the genotyping data, 28 [mut] individuals were shown to be recombinant in LG08 and the identification of the zone where the CO took place allowed to reduce the size of the mutated locus between SSR M010_3 and M010_4 (Figure 8). Therefore, this first round of genotyping on individuals allowed us to reduce the candidate mutated locus from 4.4 Mbp to 740 kb, corresponding to the distance between these two markers. Among the 48 initial recombinants, 8 were recombinant between these two markers. Additional SSR markers were designed *de novo* in this region (see section Amplification of Ectocarpus SSR markers for new SSR design and Supplementary Table 2 for oligonucleotide sequences). Out of the 15 SSR candidates found in this genomic sequence, only 4 were shown to be polymorphic (M010_3_12, M010_3_13, M010_3_14 and M010_3_15). Subsequent genotyping experiments allowed to exclude the region between M010_3 and M010_3_12, reducing the genomic window from 740 to 553 kb (Figure 8). Among the 8 individuals recombinating between M010_3 and M010_4, 4 were shown to be recombinant in the genomic region where the mutated locus should be located.

### NGS-based mapping analysis

Bulked genomic DNA of the [mut] segregative population was sequenced following the same procedure as described in section Specific knowledge and tool requirement prior to localisation of a mutant locus. The read sequences have been deposited into the European Nucleotide Archive (ENA), with the accession number PRJEB8207. Using discoSNP to identify SNPs among the short reads, we found 196,648 candidates. Among these, ≈56.6% are either purine-purine (55745) or pyrimidine-pyrimidine (55524) substitutions. These proportions are similar to those observed by comparing the two parental strains, Ec32 and Ec568 (see above and Figure 6). This shows that the UV-induced mutagenesis did not result in a huge accumulation of C/T (hence G/A on the complementary strand) mutations spread throughout the genome. We mapped the candidate SNPs onto the super-contigs of *E. siliculosus*, keeping only 97,420 unique matches with no more than one mismatch (in addition to the SNP itself). In order to eliminate spurious matches, we discarded the SNPs having a quality <55 (4216 = 4.3%) and those which were covered by less than 8 reads (7657 = 7.8%). As a result, 85613 SNP were retained for further analysis. Assuming that the distribution of the number of SNPs in a window follows a Γ law (Kendal, 2003), the mean *m* = 93.84 and standard deviation sd = 53.04 observed in 200 kb windows for the [mut] segregative population allow to estimate the parameters of the law: shape *k* = 3.13 and scale θ = 29.98.

**Figure 9:**
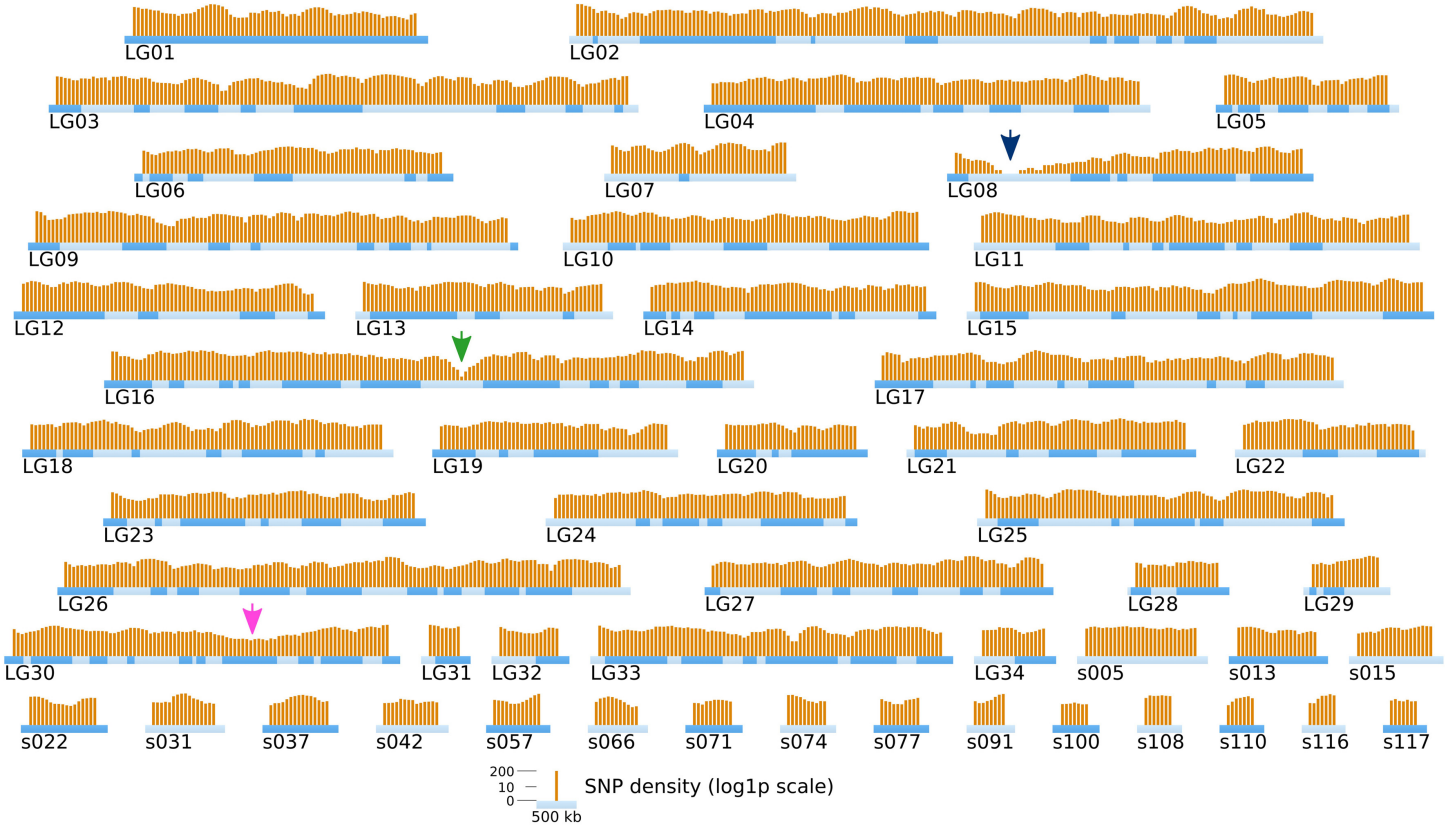
**Position of the mutated locus inferred from NGS-based mapping**. The density of SNPs computed from NGS sequencing data of the bulked gDNA of 48 offspring individuals is displayed on the genetic map of Ectocarpus (LGs and long super-contigs, see legend of Figure 5). Each orange bar represents the log1p-density in one window of 200 kb, and the shift between two successive windows is 50 kb. Green arrow, EsV1; Pink arrow, sex locus; Blue arrow, *mut* locus.

Comparison of Figures 6 and 9 shows that, in addition to the two previously identified regions of low SNP density, a new ≈600 kb region of strong SNP depletion, specific of the [mut] population, was present on the sctg10 (part of LG08): in every 200 kb window within this region, the SNP density was lower than 2/kb. Taking into account the parameters of the Γ distribution law computed above, such a low density would occur by chance with a probability *p* < 2.8 × 10^−5^ in one window, thus *p*^3^ < 2.2 × 10^−14^ for three consecutive non-overlapping windows spanning a total length of 600 kb. Similarly to the comparison between strains Ec568 and Ec32, mapping the reads from the segregative [mut] onto the Ec32 genome allowed to match a mean of 187.4 ± 17.3 reads/kb. On sctg_52 and sctg_68, the mapping led to 134.6 reads/kb (*m* − 3.05 × sd) and 130.9 reads/kb (*m* – 3.26 × sd), respectively. As before, the high variability in these regions explains the depletion of SNPs. Noticeably, as both male and female individuals of the [mut] population have been collected for this experiment, the short-read mapping efficiency on sctg_68 is less decreased than it is when comparing Ec568 to Ec32; conversely, the results on sctg_52 do not differ significantly between the 568 and F2 [mut] strains (Figure 9). On the contrary to these two regions, the [mut] population short reads mapped to the overall sctg_10 sequence at a rate close to the average: 192.2/kb (*m* + 0.28 × sd). Hence, the large SNP-depleted region in the sctg_10 cannot result from a lack of short reads mapping. Neither can the absence of SNPs indicate that this region, for one reason or another, would be exceptionally homogeneous between Ec32 and Ec568. Indeed, it does not appear as special when the comparison is carried out between the two parental types (Figure 6). Therefore, we conclude that this region is devoid of polymorphism because it is genetically linked to the [mut] phenotype, *i.e.*, it corresponds to the *mut* locus. We predict that the causal mutation must be located within the large ≈400 kb SNP-free region framed by two SNPs at positions 557813 and 964629 on the super-contig 10.

### Combination of the two approaches

**Figure 10:**
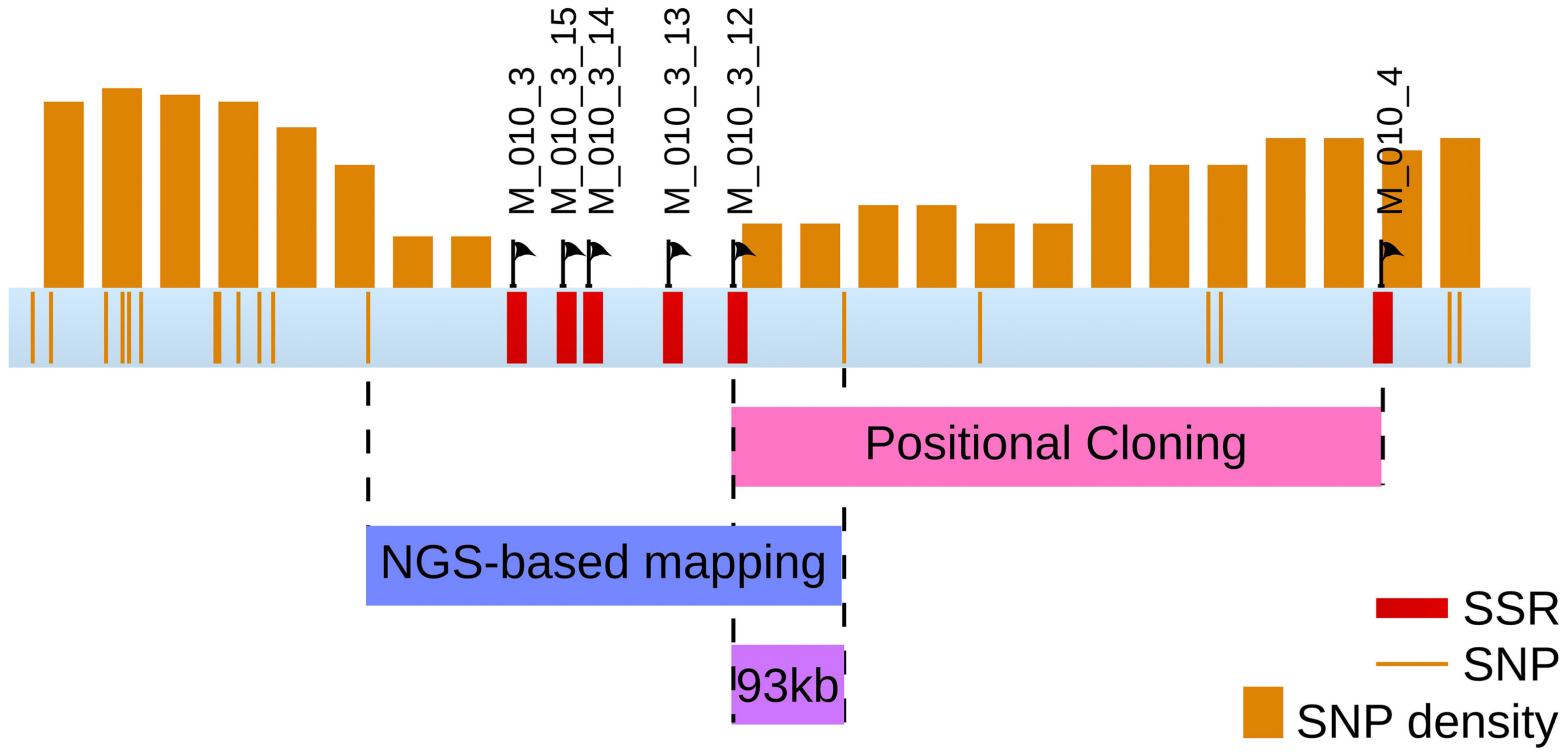
**Final localisation of the *mut* locus**. The region comprising the *mut* locus is focused from the sctg_10 super contig from LG08. Both discrete positions of SNPs and 200 kb windows are displayed, as well as the SSR markers. Overlapping the regions identified by both positional cloning and NGS-based mapping analyses allowed to select a 93 kb region containing the *mut* locus. This region contains 12 genes.

Overlapping the two regions obtained from both approaches, positional cloning and NGS-based mapping, allowed to identify a 93 kb region holding the mutated locus (Figure 10). This region contains 12 predicted genes (from Esi0010_0095 to Esi0010_0114), 7 of which with unknown predicted biological function (Cock et al., 2010). The identification of the causal mutation will subsequently be achieved by both tempting to reduce the size of the mutated locus even more, and searching for the mutation directly from the mapped NGS reads and checking by Sanger sequencing.

## Discussion

The work reported here describes how to identify a single mutation within a 214 Mbp macroalgal genome. Inspired by the experiences from land plants and metazoa, we designed a combined approach which should allow to carry out localisation of a mutated locus within less than 6 months, (2 months for the positional cloning and 1 month for the computer analyses for the NGS-based mapping) to a relatively moderate cost (Illumina NGS 85 × 10^6^ reads, + ~700 genotyping PCR). Although the experiments necessary to fully identify the mutation at the nucleotide level are beyond the scope of the present study, the combination of both approaches, positional cloning and NGS-based mapping, allowed to identify a 93 kb region carrying the mutated locus in a few weeks of work. Identifying precisely the point mutation or potential small insertion/deletion can then be performed with relatively low efforts considering the low number of genes predicted in this region.

Nevertheless, many parameters have to be considered before making this forward genetic approach handy and efficient, especially in other macroalgae. First, because these approaches rely on the capacity to identify a genomic region devoid of polymorphism (except the causal mutation itself), the size of the genomic window should be both reduced enough to contain only a few genes, and large enough to allow a reliable statement about the absence of polymorphism. Obviously, this latter requirement is directly linked to the density of the molecular markers used. Our comparison of two NGS reads (Ec568 vs. Ec32) allowed to identify ~150,000 reliable SNPs, which is much more than what can be expected from other technologies (*e.g.*, 3212 SNPs were identified in Zebrafish using Affimetrix arrays, Clark et al., 2011). In this regard, the SNPs we identified here, with a density higher than 700 Mb^−1^, constitute a promising tool for further studies, and are more valuable markers than SSRs (2 Mb^−1^).

The size of the selected genomic window also depends on the number of cross-overs added up in the overall offspring population. This is a function of both the size of the population and the recombination frequency within one specific species. In Ectocarpus, genetic analyses assessed at about 100 kb the size of the genomic region corresponding to 1 cM (54 kb in Heesch et al. (2010), and 140 kb in the present study). This allowed to calculate that ~200 individuals are to be analyzed to frame the locus in a 50 kb region. For species with a higher recombination rate, the required number of individuals will be lower. Finally the coverage rate of NGS reads also impacts the success of NGS-based mapping, as in the vicinity of the locus, a low coverage is expected for one of the SNP alleles. However, the lowest coverage rates cannot be distinguished from spurious matches known to occur when mapping short reads onto a whole genome. Therefore, by allowing to statistically assess the lesser represented reads in the pool, a global NGS coverage rate higher than the 30X used in the present study, should definitively reduce the size of the genomic window of interest.

Despite the availability of some specific protocols (Mikami, 2014), most macroalgae cannot be genetically transformed yet and forward genetic approaches still remain the only functional method to identify key genes involved in biological processes. Hence, transposition of the methods experienced herein on Ectocarpus will be of interest for these algae, provided that they are adjusted to the macroalgal species. In particular, Supercontigs assembling from NGS data is generally limited to ~ 500 kb (N50 ~ 5 kb), which, considering the high level of variation in the SNP density over the Ectocarpus genome (Figure 6) is not large enough to encompass a genomic window devoid of polymorphism. Therefore, perspectives of transposition of this method to other macroalgae should take into consideration the necessity to rely on a dense genetic map, or to use a sexual partner genetically distant enough to display a higher density of SNPs. These propositions could even be applicable to Ectocarpus, as the localisation of the *mut* locus could have been unsuccessful since only 65% of the genome is assembled into the genetic map so far. In addition, most of the super-contigs therein are un-orientated, and are represented as consecutive while they are actually separated by DNA of unknown length (which might correspond to known super-contigs not yet included in a LG). Beside the probability that the gene of interest may belong to the part of the genome which is not sequenced yet (about 10% in *E. siliculosus*), the lack of continuity between super-contigs is likely to blur the analyses relying on the genetic map. Therefore, for mutations located in the other 35% of the genome, a much higher SNP density would be required for identifying sctgs devoid of polymorphism and hence to identify the mutation.

In all cases, gene mapping approaches rely on a good knowledge of the genome organization and especially of the coding sequence. Our recent characterisation of an other Ectocarpus mutant showed that the mutation was located in a gene spanning two small sctg not assembled into any LG, and which gene structure and coding sequence were incorrectly predicted. Therefore, despite the present demonstration of the easiness of locating a mutated locus in Ectocarpus with the current tools, improving both the genomic sequence and the genetic map of Ectocarpus is necessary before considering this approach in routine.

### Conflict of interest statement

The authors declare that the research was conducted in the absence of any commercial or financial relationships that could be construed as a potential conflict of interest.
